# 

**Published:** 1891-11

**Authors:** 


### Ohio State Dental Society.

The prospects for a large and profitable meeting at Columbus, Dec. 1st, 2d, 3d, are most flattering.

The Executive Committee have spared no pains to this end.

The Sessions will be held in the new and magnificent "Hotel Chittenden," where special rates and conveniences are offered for all in attendance upon this occasion.

Papers will be presented by Drs. C. M. Wright, J. Taft, H. A. Smith, G. H. Junkerman, W. Taft, E. H. Roffensperger, J. R. Callahan, F. W. Sage, J. E. Robinson. Also Drs. A. W. Harlan, of Chicago; A. O. Rawls, of Kentucky; W. S. Elliott, of New York; and Geo. W. Melotte, of New York.

Clinical demonstrations of cases will be a conspicuous feature of the meeting, among which will be, "Glass fillings," "Inlays," Gold tippings," "Combination fillings," "Crown and Bridge work." Also exhibits of electrical appliances and other auxilliaries for dental practice.

The meeting opens promptly at 9 A. M., Tuesday, Dec. 1st.

A banquet will be given Wednesday evening at "Hotel Chittenden," to which every member and foreign guest is cordially invited. Programs will soon be distributed to the profession by mail.

Every reputable dentist should attend this meeting, where he will be made welcome.

OTTO ARNOLD, *Sec'y.*, Columbus.

### EDITORIAL.

THE DENTAL REGISTER,

A MONTHLY MAGAZINE DEVOTED TO THE INTERESTS OF THE

**DENTAL PROFESSION.**

*Terms Reduced to $2.00 Per Year. Single Copies, 25 Cents.*

**EDITED BY PROF. J. TAFT, D.D.S.**

—AND—

**Published by SAM'L A. CROCKER & CO., Ohio Dental and Surgical Depot.**

The volume commences in January and closes in December.

The REGISTER being issued monthly is always fresh, therefore Indispensable to the practitioner who desires to keep posted up with the new inventions and improvements which are daily developed. Neither expense nor effort will be spared to give value and variety to its contents Articles will be illustrated with well executed engravings whenever they will add to the interest or assist to explanation.

Its extensive circulation and frequency of issue make it one of the BEST DENTAL ADVERTISING MEDIUMS in the United States.

All subscriptions must begin with January or July.

Local or special notices, five lines or less, will be inserted for $3 each insertion; over five lines, 50 cts. per line.

All advertising bills payable in advance, or at furthest, after the issue of the first number containing the advertisement. All transient advertisements to be paid for in advance.

☞CONTRIBUTIONS SOLICITED.

Exclusively Editorial Communications should be addressed to the Editor.

The latest number of the REGISTER may be ordered with or without other goods at any time, and all letters should be addressed to

SAM'L A. CROCKER & CO., Ohio Dental and Surgical Depot, Cincinnati, O.

